# Supplementary material for: Assessing the effectiveness of malaria interventions at the regional level in Ghana using a mathematical modelling application
Source: PLOS Glob Public Health. 2022 Dec 21;2(12):e0000474. doi: 10.1371/journal.pgph.0000474 (PMC10021332; doi:10.1371/journal.pgph.0000474)
Supplement: S1 Table — (DOCX) [file pgph.0000474.s001.docx]

# S1 Table: Tables

# Table A Parameters for forcing functions by zone

| Zone | T1 | T2 | T3 | T4 | $\boldsymbol{\emptyset}_{\boldsymbol{1}}$ | $\boldsymbol{\emptyset}_{\boldsymbol{2}}$ | pe |
| --- | --- | --- | --- | --- | --- | --- | --- |
| Guinea savannah | 0.5425 | 0.4510 | - | - | 16.2019 | - | 2 |
| Transitional forest | -11.0593 | 0.8012 | 10.8939 | 0.3329 | 4.7505 | 8.5291 | 2 |
| Coastal savannah | 48.5421 | 0.7323 | -48.7133 | 0.2802 | -1.2691 | -179.8410 | 2 |

# Interventions deployed singly

# Table B Predictions of cases averted (%) and incidence/1000 population for regions of the Guinea savannah

| Region | CHW | | HSS | | ITN | | IRS | | SMC | |
| --- | --- | --- | --- | --- | --- | --- | --- | --- | --- | --- |
|  | Cases averted | Incidence | Cases averted | Incidence | Cases averted | Incidence | Cases averted | Incidence | Cases averted | Incidence |
| Upper East | 0.9 | 377 | -21.7 | 466 | 9.3 | 337 | 5.5 | 350 | 5.6 | 353 |
| Upper West | 0 | 321 | -41.9 | 472 | 7.8 | 294 | 0.4 | 319 | 5.4 | 304 |
| Northern | 0.1 | 143 | 61.7 | 19 | 79.3 | 13 | 51.3 | 50 | 40.8 | 80 |

# Table C Predictions of cases averted (%) and incidence/1000 population for regions of the Transitional forest

| Region | CHW | | HSS | | IRS | | ITN | |
| --- | --- | --- | --- | --- | --- | --- | --- | --- |
|  | Cases averted | Incidence | Cases averted | Incidence | Cases averted | Incidence | Cases averted | Incidence |
| Ashanti | 0.1 | 141 | 53.6 | 37 | 48.2 | 57 | 67.9 | 31 |
| Brong-Ahafo | -0.2 | 286 | -36.9 | 403 | 10.5 | 252 | 18.3 | 228 |
| Eastern | -0.1 | 212 | 36.8 | 74 | 37.1 | 116 | 67.5 | 43 |
| Volta | 0.1 | 170 | 52.7 | 47 | 52.2 | 59 | 72.5 | 34 |

# Table D Predictions of cases averted (%) and incidence/1000 population for regions of the Coastal savannah

| Region | CHW | | HSS | | IRS | | ITN | |
| --- | --- | --- | --- | --- | --- | --- | --- | --- |
|  | Cases averted | Incidence | Cases averted | Incidence | Cases averted | Incidence | Cases averted | Incidence |
| Central | -1.1 | 223 | -4.1 | 195 | 26.0 | 154 | 47.4 | 91 |
| Greater Accra | 1.1 | 44 | 64.0 | 9 | 69.1 | 8 | 85.4 | 4 |
| Western | 0.1 | 249 | -24.3 | 311 | 19.6 | 194 | 40.6 | 129 |

# Interventions deployed in combination

# Table E Predictions of cases averted and incidence/1000 population for regions of the savannah zone

| Region | CHW + ITN | | HSS + ITN | | ITN + IRS | | ITN + SMC | |
| --- | --- | --- | --- | --- | --- | --- | --- | --- |
|  | Cases reported (%) | Incidence | Cases reported (%) | Incidence | Cases averted (%) | Incidence | Cases averted (%) | Incidence |
| Upper East | 68.9 | 44 | 76.0 | 14 | 86.4 | 6 | 75.4 | 27 |
| Upper West | 44.6 | 157 | 59.3 | 34 | 71.0 | 44 | 52.2 | 129 |
| Northern | 95.8 | 1.4 | 95.4 | 1.7 | 96.6 | 0.9 | 96.4 | 1.2 |

# Table F Predictions of cases averted and incidence/1000 population for regions of the Transitional forest zone

| Region | CHW + ITN | | HSS + ITN | | ITN + IRS | |
| --- | --- | --- | --- | --- | --- | --- |
|  | Cases averted | Incidence | Cases averted | Incidence | Cases averted | Incidence |
| Ashanti | 83.0 | 20 | 82.6 | 20 | 83.9 | 20 |
| Brong-Ahafo | 80.2 | 31 | 80.9 | 31 | 84.4 | 29 |
| Eastern | 86.9 | 22 | 86.8 | 22 | 87.7 | 21 |
| Volta | 83.9 | 25 | 83.8 | 25 | 84.3 | 25 |

# Table G Predictions of cases averted and incidence/1000 population for regions of the Coastal savannah zone

| Region | CHW + ITN | | HSS + ITN | | ITN + IRS | |
| --- | --- | --- | --- | --- | --- | --- |
|  | Cases averted | Incidence | Cases averted | Incidence | Cases averted | Incidence |
| Central | 91.6 | 2 | 91.6 | 3 | 94.4 | 1 |
| Greater Accra | 95.2 | 1 | 94.4 | 1 | 96.3 | 1 |
| Western | 92.1 | 2 | 92.1 | 3 | 94.7 | 1 |
