# Supplementary material for: Assessing the effectiveness of malaria interventions at the regional level in Ghana using a mathematical modelling application
Source: PLOS Glob Public Health. 2022 Dec 21;2(12):e0000474. doi: 10.1371/journal.pgph.0000474 (PMC10021332; doi:10.1371/journal.pgph.0000474)
Supplement: S2 Table — (DOCX) [file pgph.0000474.s002.docx]

| S2 Table: Table of Parameters and their source by region for model application | | | | | | | | | | | |
| --- | --- | --- | --- | --- | --- | --- | --- | --- | --- | --- | --- |
| **Zone** | **Savannah** | | | **Forest** | | | | **Coastal** | | | **Source** |
| **Region** | **Upper East** | **Upper West** | **Northern** | **Ashanti** | **Bono-Ahafo** | **Eastern** | **Volta** | **Central** | **Greater Accra** | **Western** |  |
| Population | 1300000 | 850000 | 3062000 | 5800000 | 2630000 | 3200000 | 2613000 | 2600000 | 5000000 | 3400000 | [1] |
| Annual population growth rate | 1.2 | 1.9 | 2.9 | 2.7 | 2.3 | 2.1 | 2.5 | 3.1 | 3.1 | 2.0 | [1] |
| Average number of people per village | 1000 | 1000 | 1000 | 1000 | 1000 | 1000 | 1000 | 1000 | 1000 | 1000 | expert |
| API/1000 |  |  |  |  |  |  |  |  |  |  | [2] |
| 2012 | 230 | 129 | 81 | 66 | 102 | 112 | 67 | 92 | 44 | 84 |  |
| 2013 | 286 | 188 | 91 | 90 | 150 | 148 | 104 | 110 | 40 | 118 |  |
| 2014 | 230 | 325 | 88 | 90 | 235 | 142 | 115 | 129 | 44 | 134 |  |
| 2015 | 285 | 257 | 118 | 102 | 241 | 208 | 132 | 178 | 47 | 188 |  |
| 2016 | 381 | 245 | 116 | 111 | 228 | 194 | 160 | 173 | 46 | 201 |  |
| 2017 | 371 | 237 | 150 | 111 | 248 | 181 | 162 | 203 | 50 | 231 |  |
| 2018 | 371 | 329 | 150 | 142 | 291 | 214 | 170 | 224 | 48 | 254 |  |
| Imported cases/1000 | 10 | 10 | 10 | 1 | 150 | 150 | 150 | 50 | 50 | 150 | expert |
| Multiplier | 1.5 | 1.5 | 1.5 | 1.5 | 1.5 | 1.5 | 1.5 | 1.5 | 1.5 | 1.5 | expert |
| HIS: Diagnosis and non-malaria (proportion) | 0.66 | 0.66 | 0.66 | 0.66 | 0.66 | 0.66 | 0.66 | 0.66 | 0.66 | 0.66 | expert |
| RDT positivity rate | 0.45 | 0.34 | 0.38 | 0.29 | 0.32 | 0.4 | 0.31 | 0.37 | 0.21 | 0.48 | [3] |
| Slide positivity rate | 0.38 | 0.23 | 0.38 | 0.45 | 0.18 | 0.3 | 0.2 | 0.21 | 0.15 | 0.47 |  |
| Probability of seeking treatment at Health Facility | 0.89 | 0.73 | 0.68 | 0.67 | 0.68 | 0.75 | 0.68 | 0.62 | 0.81 | 0.66 | [4] |
| Probability of being tested at Health Facility | 0.90 | 0.85 | 0.58 | 0.70 | 0.79 | 0.82 | 0.84 | 0.90 | 0.85 | 0.85 | [3] |
| Probability of being treated at Health Facility after a positive test | 0.56 | 0.61 | 0.50 | 0.48 | 0.59 | 0.59 | 0.66 | 0.55 | 0.34 | 0.60 |  |
| Probability of cases reported | 0.90 | 0.90 | 0.90 | 0.90 | 0.90 | 0.90 | 0.90 | 0.90 | 0.90 | 0.90 | NMCP |
| Sensitivity of RDT- Clinical | 0.56 | 0.56 | 0.56 | 0.56 | 0.56 | 0.56 | 0.56 | 0.56 | 0.56 | 0.56 | [5] |
| Microscopy undetectable, Asymptomatic | 0.05 | 0.05 | 0.05 | 0.05 | 0.05 | 0.05 | 0.05 | 0.05 | 0.05 | 0.05 | expert |
| Treatment failure | 0.05 | 0.05 | 0.05 | 0.05 | 0.05 | 0.05 | 0.05 | 0.05 | 0.05 | 0.05 | expert |
| Community Health Worker(CHW/CHPS) | 0.024 | 0.086 | 0.087 | 0.05 | 0.013 | 0.050 | 0.026 | 0.001 | 0.003 | 0.011 | [6] |
| Seek treatment with (CHW/CHPS) | 0.769 | 0.769 | 0.769 | 0.363 | 0.363 | 0.363 | 0.363 | 0.363 | 0.363 | 0.363 | [7] |
| Being tested with (CHW/CHPS) | 0.417 | 0.417 | 0.417 | 0.653 | 0.653 | 0.653 | 0.653 | 0.653 | 0.653 | 0.653 |  |
| Being treated after positive (CHW/CHPS) | 0.359 | 0.359 | 0.359 | 0.155 | 0.155 | 0.155 | 0.155 | 0.155 | 0.155 | 0.155 |  |
| Cases reported from (CHW/CHPS) | 0.93 | 0.93 | 0.93 | 0.93 | 0.93 | 0.93 | 0.93 | 0.93 | 0.93 | 0.93 | [8] |
| CHW/CHPS coverage |  |  |  |  |  |  |  |  |  |  |  |
| 2012** | 0.165 | 0.138 | 0.221 | 0.400 | 1.00 | 0.500 | 0.029 | 0.083 | 0.400 | 0.102 | [3,9] |
| 2013** | 0.308 | 0.318 | 0.233 | 0.032 | 0.060 | 0.357 | 0.126 | 0.222 | 0.022 | 0.223 | [3,10] |
| 2014 | 0.638 | 0.512 | 0.400 | 0.664 | 0.497 | 0.527 | 0.405 | 0.399 | 0.151 | 0.354 | [8] |
| 2015** | 0.698 | 0.622 | 0.430 | 0.818 | 0.553 | 0.540 | 0.421 | 0.422 | 0.187 | 0.420 | [3,11] |
| 2016 | 0.841 | 0.770 | 0.677 | 0.910 | 0.729 | 0.711 | 0.629 | 0.491 | 0.293 | 0.750 | [3] |
| 2017 | 0.841 | 0.770 | 0.677 | 0.910 | 0.729 | 0.711 | 0.629 | 0.491 | 0.293 | 0.750 |  |
| 2018 | 0.760 | 0.904 | 100 | 0.987 | 0.817 | 0.844 | 0.680 | 0.559 | 0.319 | 0.943 | [12] |
| **ITN-** Effectiveness | 0.252 | 0.216 | 0.204 | 0.172 | 0.208 | 0.156 | 0.184 | 0.2 | 0.072 | 0.148 | [13,14] |
| ITN distribution/universal coverage* |  |  |  |  |  |  |  |  |  |  |  |
| 2012 | 0.177 | 0.194 | 0.193 | 0.161 | 0.214 | 0.573 | 0.652 | 0.104 | 0.126 | 0.214 | [15] |
| 2013 | 0.177 | 0.194 | 0.193 | 0.161 | 0.214 | 0.573 | 0.652 | 0.104 | 0.126 | 0.214 | [16] |
| 2014 | 0.354 | 0.383 | 0.338 | 0.400 | 0.539 | 0.458 | 0.540 | 0.313 | 0.408 | 0.409 | [16] |
| 2015 | 0.352 | 0.383 | 0.338 | 0.400 | 0.539 | 0.458 | 0.540 | 0.313 | 0.408 | 0.409 | [17] |
| 2016 | 0.881 | 0.767 | 0.767 | 0.599 | 0.721 | 0.605 | 0.664 | 0.539 | 0.761 | 0.591 | [17] |
| 2017 | 0.736 | 0.682 | 0.682 | 0.589 | 0.637 | 0.596 | 0.653 | 0.523 | 0.627 | 0.574 | [18] |
| 2018 | 0.736 | 0.682 | 0.682 | 0.589 | 0.637 | 0.596 | 0.653 | 0.523 | 0.627 | 0.574 | [18] |
| ITN Coverage at 2018 (%) | **74** | **74** | **68** | **59** | **64** | **60** | **65** | **52** | **63** | **57** | Estimated from coverage |
| IRS-Coverage (%) |  |  |  |  |  |  |  |  |  |  |  |
| 2012 | 0 | 36.7 | 30.7 | 4.5 | 0 | 0 | 0 | 1 | 0 | 0 | [19,20] |
| 2013 | 40.4 | 70.5 | 17.4 | 6.6 | 0 | 0 | 0 | 3.8 | 0 | 11.2 |  |
| 2014 | 41.6 | 26.9 | 18.6 | 3.7 | 0 | 0 | 0 | 4.6 | 0 | 10.4 |  |
| 2015 | 0 | 76.3 | 18.1 | 2.1 | 0 | 0 | 0 | 0 | 0 | 0 |  |
| 2016 | 0 | 84.6 | 18.6 | 2.1 | 0 | 0 | 0 | 0 | 0 | 0 |  |
| 2017 | 13.5 | 84.7 | 27.4 | 2.3 | 0 | 0 | 0 | 0 | 0 | 0 |  |
| 2018 | 13.5 | 84.6 | 27.3 | 2.1 | 0 | 0 | 0 | 0 | 0 | 0 |  |
| Infections averted due to IRS | 0.3 | 0.3 | 0.3 | 0.3 | 0.3 | 0.3 | 0.3 | 0.3 | 0.3 | 0.3 | [13] |
| Coverage of IRS programme | 0 |  |  |  |  |  |  |  |  |  |  |
| Active Case detection | 0 | 0 | 0 | 0 | 0 | 0 | 0 | 0 | 0 | 0 |  |
| Active Screening | 0 | 0 | 0 | 0 | 0 | 0 | 0 | 0 | 0 | 0 |  |
| SMC | 0.1 | 0.1 | 0 | 0 | 0 | 0 | 0 | 0 | 0 | 0 | NMCP |
| **IPTp-** Startyear | 2012 | 2012 | 2012 | 2012 | 2012 | 2012 | 2012 | 2012 | 2012 | 2012 | year |
| TFR | 4.7 | 4.6 | 5.8 | 3.8 | 4.0 | 3.8 | 4.1 | 4.3 | 2.8 | 4.1 | [21] |
| ANC registration | 0.64 | 0.64 | 0.64 | 0.64 | 0.64 | 0.64 | 0.64 | 0.64 | 0.64 | 0.64 | [21] |
| **IPTp Coverage** |  |  |  |  |  |  |  |  |  |  |  |
| IPTp 1 | 0.60 | 0.51 | 0.61 | 0.6 | 0.8 | 0.65 | 0.7 | 0.65 | 0.62 | 0.6 | [3] |
| IPTp 2 | 0.45 | 0.4 | 0.41 | 0.51 | 0.65 | 0.52 | 0.55 | 0.52 | 0.52 | 0.48 |  |
| IPTp 3 | 0.32 | 0.24 | 0.27 | 0.48 | 0.5 | 0.4 | 0.36 | 0.4 | 0.41 | 0.32 |  |
| IPTp 4 | 0.18 | 0.08 | 0.11 | 0.18 | 0.22 | 0.2 | 0.17 | 0.2 | 0.18 | 0.15 |  |
| IPTp 5 | 0.05 | 0.02 | 0.05 | 0.08 | 0.08 | 0.08 | 0.05 | 0.08 | 0.08 | 0.05 |  |
| Pf transmission | 500 | 1500 | 500 | 130 | 350 | 97 | 95 | 380 | 93.5 | 600 | Simulation |
| Case Fatality Rate | 0.51 | 0.35 | 0.49 | 0.13 | 0.26 | 0.23 | 0.39 | 0.36 | 0.29 | 0.32 | [3] |
| Treatment seeking for severe illness | 0.95 | 0.95 | 0.95 | 0.95 | 0.95 | 0.95 | 0.95 | 0.95 | 0.95 | 0.95 |  |
| Amplitude 1 | 1 | 1 | 0.65 | 0.72 | 1 | 0.85 | 0.85 | 1 | 0.82 | 1 | Estimated from data |
| Amplitude 2 | 1 | 1 | 0.50 | 0.68 | 1 | 0.88 | 0.80 | 0.46 | 0.30 | 0.57 |  |
| Peak month 1 | 10 | 10 | 10 | 6 | 6 | 6 | 6 | 7 | 7 | 7 |  |
| Peak month 2 | - | - | - | 10 | 10 | 10 | 10 | 10 | 10 | 10 |  |
| Peakedness | 3 | 4 | 2 | 2 | 2 | 2 | 2 | 2 | 2 | 2 |  |

# * LLINs coverage levels from GHS reports

# ** 2012, 2013 and 2015 facts and figures using CHPS table in 2016 GHS report as reference

# References

1. Regional population of Ghana [Internet]. [cited 2021 Jun 9]. Available from: https://statsghana.gov.gh/regionalpopulation.php?population=MTUwNDMxMDk2MS40NjA1&&UpperWest&regid=9

2. Adaletey DL, Jolliffe B, Braa J, Ofosu A. Peer-performance review as a strategy for strengthening health information systems: A case study from Ghana. J Health Inform Afr. 2014;2:103.

3. Ghana Health Service (GHS). Ghana Health Service report, 2016 [Internet]. 2016. Available from: https://www.ghanahealthservice.org/downloads/GHS_ANNUAL_REPORT_2016_n.pdf

4. Ghana Health Service (GHS). 2017 Annual report, National malaria control programme. 2018.

5. Dinko B, Ayivor R, Abugri J, Agboli E, Kye G, Tagboto S, et al. Comparison of malaria diagnostic methods in four hospitals in the Volta region of Ghana. 2016;7:7.

6. Ghana Statistical Service. Ghana Malaria Indicator Survey 2016 [Internet]. Ghana Statistical Service; 2017 [cited 2017 Nov 4]. Available from: http://www.statsghana.gov.gh/docfiles/publications/Ghana%20MIS%202016%20KIR%20-%2006March2017.pdf

7. Ferrer BE, Webster J, Bruce J, Narh- Bana SA, Narh CT, Allotey N-K, et al. Integrated community case management and community-based health planning and services: a cross sectional study on the effectiveness of the national implementation for the treatment of malaria, diarrhoea and pneumonia. Malar J. 2016;15:340.

8. Ghana Health Service (GHS). Ghana Health Service report 2014 [Internet]. 2014. Available from: https://www.ghanahealthservice.org/ghs-item-details.php?cid=2&scid=52&iid=107

9. Ghana Health Service (GHS) DE. The Health Sector in Ghana: Facts and Figures. 2012;48.

10. Ghana Health Service (GHS) DE. The Health Sector in Ghana: Facts and Figures. 2013;46.

11. Ghana Health Service (GHS). The Health Sector in Ghana: Facts and Figures. 2015;55.

12. Ghana Health Service (GHS), Research and Development Division. CHPS verification survey. :248.

13. Kesteman T, Randrianarivelojosia M, Rogier C. The protective effectiveness of control interventions for malaria prevention: a systematic review of the literature. F1000Research. 2017;6:1932.

14. Hannah Koenker, Emily Ricotta, Bolanle Olapeju, Ifta Choiriyyah. Insecticide-Treated Nets (ITN) Access and Use Report.Baltimore, MD. PMI | VectorWorks Project, Johns Hopkins Center for Communication Programs. 2018.

15. Ghana Statistical Service. Ghana Multiple Indicator Cluster Survey with an Enhanced Malaria Module and Biomaker,2011, Final report. [Internet]. Ghana Statistical Service; 2011 [cited 2015 Dec 23]. Available from: http://www.unicef.org/ghana/Ghana_MICS_Final.pdf

16. Ghana Statistical Service. Ghana Demographic and Health Survey,2014 [Internet]. Ghana Statistical Service; 2014 [cited 2015 Dec 23]. Available from: http://dhsprogram.com/what-we-do/survey/survey-display-437.cfm

17. Ghana Statistical Service (GSS), Ghana Health Service (GHS). Ghana Malaria Indictor Survey, 2016 [Internet]. Accra, Ghana, and Rockville, Maryland, USA: GSS, GHS, and ICF; 2017. Available from: www.DHSprogram.com

18. Ghana Statistical Service. Multiple Indicator Cluster Survey (MICS2017/18), Survey Findings Report. 2018.

19. Ghana End of Spray Report 2017. :90.

20. Agamal Ltd. Agamal IRS coverage by region and year.

21. Ghana Statistical Service, Ghana Health Service (GHS), ICF. Ghana Maternal Health Survey 2017: Key Findings [Internet]. 2018 [cited 2020 Jun 12]. Available from: https://www.dhsprogram.com/pubs/pdf/SR251/SR251.pdf
