# Supplementary material for: Assessing the effectiveness of malaria interventions at the regional level in Ghana using a mathematical modelling application
Source: PLOS Glob Public Health. 2022 Dec 21;2(12):e0000474. doi: 10.1371/journal.pgph.0000474 (PMC10021332; doi:10.1371/journal.pgph.0000474)
Supplement: Attachment — Submitted filename: Reviewer_comments_answers.docx [file pgph.0000474.s003.docx]

Journal Requirements:

1. Please amend your detailed Financial Disclosure statement. This is published with the article. It must therefore be completed in full sentences and contain the exact wording you wish to be published.

a. State the initials, alongside each funding source, of each author to receive each grant.

**Response: The statement was mended to read “SPS was funded by the Wellcome Trust (GN: 2114236/Z/18Z) to develop the SPPf Application. This study is part of TA’s PhD studies sponsored by SACEMA and the Ghana Education Trust Fund (GetFund). The funders had no role in study design, data collection and analysis, decision to publish, or preparation of the manuscript”.**

b. State what role the funders took in the study. If the funders had no role in your study, please state: “The funders had no role in study design, data collection and analysis, decision to publish, or preparation of the manuscript.”

**Response: “The funders had no role in study design, data collection and analysis, decision to publish, or preparation of the manuscript” has been added to the funding statement as suggested.**

2. Please update your online Competing Interests statement. If you have no competing interests to declare, please state: “The authors have declared that no competing interests exist.”

**Response: Competing Interests statement has been updated with “The authors have declared that no competing interests exist.”**

3. We ask that a manuscript source file is provided at Revision. Please upload your manuscript file as a .doc, .docx, or .rtf.

**Response: Draft manuscript is save in doc format**

4. Please ensure that you refer to Fig 6 in your text as, if accepted, production will need this reference to link the reader to the figure.

**Response: Link to Fig 6 has been made through changes to Fig 5 in the last sentence before the caption for the figure**

5. Please include a copy of Table 2 which you refer to in your text on page 6.

**Response: Table 2 changed to S3 Table**

6. We have noticed that you have uploaded Supporting Information files, but you have not included a list of legends. Please add a full list of legends for your Supporting Information files after the references list.

**Response: List of legends for supporting documents has been provided**

Reviewer #1: Great to see this work

1. Although the initial model is based on an already in use model which as a strength avoids cumbersome implementation and jargon [https://www.ncbi.nlm.nih.gov/pmc/articles/PMC6971843/](https://www.ncbi.nlm.nih.gov/pmc/articles/PMC6971843), it is still important to discuss validation further. As a model is only as good as its assumptions, it would be best to discuss caveats, potential pitfalls, and any validation or plans to validate. The modification to the seasonal forcing functions to account for the seasonal spikes could potentially have different impacts. Policy makers, though often wanting a straightforward tool without a lat of jargon, do want to see data and validation so any form of validation (on past data) or plans to validate would still be useful for policy makers.

- I would add any validation run on past data or plans for future validation or potential foreseen caveats to the discussion

**Response: The model was calibrated and validated using monthly data from different transmission settings in Ghana. Data each transmission zone from 2012 to 2017 years were used to calibrate the model. Each zonal model was then validated using data from 2017 for a year. As shown in the description file in** <https://gitlab.com/tawine/SPPf_tool_Ghana_regional_analysis>**,the seasonal patterns of malaria incidence in the various zones have been captured and 50% projected uncertainty range captures most of the observed data as well within the validation range**

- I would focus more on discussing the model itself and its strengths but also potential than the specific outputs

**Response: Key features of malaria epidemiology in an endemic country such as Ghana have been taken account of in the model. These features include superinfection and immunity. Given the high levels of malaria incidence in all three zones, the model outputs may be greatly improved having these key features incorporated even though the full knowledge of how immunity works is not yet not understood[16]. Additionally the model accounts for the various stages of malaria infection and makes room for diagnostic sensitivity for these classes of infection. It incorporates adjustments for a possible El Nino event and provides the opportunity to be adapted for various transmission settings through the incorporation of monthly rainfall for a given location of interest.**

2. It would be helpful to discuss how other models have included seasonality. Is it possible to compare with other models that have looked at seasonality?
[https://www.ncbi.nlm.nih.gov/pmc/articles/PMC9167503/](https://www.ncbi.nlm.nih.gov/pmc/articles/PMC9167503)

**Response: The seasonal patterns have been used in other models for using the same data for calibration. However, comparison will be difficult to undertake as the previous models investigated the impact of various interventions at a macro level (zonal) whiles the focus in the present model is at the regional administrative level. The other model is:** **https://malariajournal.biomedcentral.com/articles/10.1186/s12936-020-03267-9**

3. It would be helpful if making predictions into the 2030s to include discussion, at least, of other factors which may affect continuing progress and could discuss other
- Potential artesunate resistance (though not currently a pressing issue could reach a tipping point where becomes a factor in the next decade [https://pubmed.ncbi.nlm.nih.gov/33746510/](https://pubmed.ncbi.nlm.nih.gov/33746510)<https://www.nature.com/articles/s41598-022-11790-9>
<https://www.nejm.org/doi/full/10.1056/NEJMoa2101746>
<https://www.who.int/news-room/questions-and-answers/item/artemisinin-resistance>

- ITN resistance, though often overstated, still worth mentioning [https://pubmed.ncbi.nlm.nih.gov/35331237/](https://pubmed.ncbi.nlm.nih.gov/35331237)

- Likewise IRS resistance as have needed to change in the past, though not anticipated now could have resistance more so in the coming decade(s)[https://www.ncbi.nlm.nih.gov/pmc/articles/PMC7353711/](https://www.ncbi.nlm.nih.gov/pmc/articles/PMC7353711)

**Response: A statement has been added on the predictions into the future and development of insecticide resistance in Ghana to read “The predictions made into the future to 2030 were based on the assumptions that, current environment and vector behaviour remains constant going into the future. With respect to vector behaviour, recent studies have suggested an emerging threat of insecticide resistance particularly for pyrethroids in some parts of Ghana. Unfortunately the use of this insecticide for both pests and vector control form part of the main strategies of vector control activities through treated bed nets and IRS. The impact of this development may be minimised or averted if alternate insecticides are deployed for both treated bed nets and IRS [12,13]. This notwithstanding, the use of treated bednets and IRS may still proffer some protection to the larger rural population once they are used effectively[11,14,15]”.**

**.**

**.**

- pfhrp2/3 gene deletions (and false negative RDTs) which is a growing problem in Africa particularly the Horn of Africa, and has not proven itself much of an issue at this time in Ghana but pathogens are dynamic and mobile, so over the course of decade this could change.
<https://www.who.int/news/item/28-05-2021-statement-by-the-malaria-policy-advisory-group-on-the-urgent-need-to-address-the-high-prevalence-of-pfhrp2-3-gene-deletions-in-the-horn-of-africa-and-beyond>
<https://www.who.int/publications/i/item/WHO-CDS-GMP-2019.02>
<https://journals.plos.org/plosone/article?id=10.1371/journal.pone.0238749>
<https://malariajournal.biomedcentral.com/articles/10.1186/s12936-019-2987-4>

- Climate Change. Although the model includes El Niño and weather information, I would still make note of how climate change which may disrupt current patterns and cause greater drought, seasonal changes, even population movement away from say dry areas to wetter areas, could affect predictions in the longerterm rather than just a year or so in advance. The modification of seasonal forcing functions could behave differently if climate change acerbated.

**Response: The model allows for changes to be made to the forcing function as this will be captured in the weather data used for fitting the models. This has been included in the statement above about changes in the environment and its impact on the predictions.**

- Mobility patterns can also affect the number in certain zones and also the immunological landscape.

**Response: We assumed that, level of malaria endemicity across Ghana will hardly be affected by mobility across regions or zones. Similarly, the population is constantly being exposed and therefore the levels of immunity of the populations in different regions should not vary to be concerned about.**

Minor
Line Numbers would help a lot.

**Response: edited to include line numbers**

Do ensure abbreviations are defined on first use, even if commonly understood (IRS) to allow readers outside of field or sub-field to appreciate paper

**Response: edited appropriately**

Check use of it's vs its
**Response: edited appropriately**

Also 404 message on gitlab when logged in:
[https://gitlab.com/tawine/SPPf_tool_Ghana_regional_analysis](https://gitlab.com/tawine/SPPf_tool_Ghana_regional_analysis)

**Response: The link in Github has been made public and therefore accessible by all currently.**

Reviewer #2: This is an important article to help in planing for malaria elimination and also relevant in overall disease control planning and forecasting.
I would suggest the meaning of the acronym SMC is clarified as it is rather a chemo prevention strategy and not a chemotherapy. I also suggest that the acronyms ITN is spelt out in full at the first mention.
It may also be better to be consistent with either using insecticide treated nets or long lasting insecticide treated nets or bednets in the abstract and main article, with a clear definition of what it refers to.
I am.also of the view that the cost effectiveness of each strategy may have been useful to help in decision making. Authors did mention that a study had looked into the cost of different strategies. The discussion may have highlighted the potential cost of each modelled outcomes

**Response: SMC as a chemo preventive strategy has been corrected as suggested to read “Seasonal Malaria Chemoprevention”. ITN has been written fully as in it appears for the first time. LLINs has been replaced with ITN for consistency as suggested.**

**Cost effectiveness of the interventions on zonal basis using the regions as aggregates, have earlier on been investigated and published as In** [**https://doi.org/10.1186/s12936-020-03267-9**](https://doi.org/10.1186/s12936-020-03267-9)**. We did not want to duplicate the results here but a statement has been made to refer readers to the cost implications of various interventions considered.**

Reviewer #3: The language used in the submitted article is clear, coherent and concise. The model application employed for the study is logical and convincing. The method employed tor the study is appropriate. All tables are relevant to the method and the objectives of the study. The discussion section was appropriate and sufficient. The limitation was mentioned in a justifiable way, e.g., inter-regional coverage and age distribution.

**Response: No comments**
