# Supplementary material for: Assessing the effectiveness of malaria interventions at the regional level in Ghana using a mathematical modelling application
Source: PLOS Glob Public Health. 2022 Dec 21;2(12):e0000474. doi: 10.1371/journal.pgph.0000474 (PMC10021332; doi:10.1371/journal.pgph.0000474)
Supplement: Attachment — Submitted filename: Reviewers comments.docx [file pgph.0000474.s004.docx]

Reviewers' comments:

Dear Editor,

Thanks for the comments. The answers to the comments are marked in blue as in the responses below.

Best wishes.

Timothy Awine

Reviewer's Responses to Questions

**Comments to the Author**

1. If the authors have adequately addressed your comments raised in a previous round of review and you feel that this manuscript is now acceptable for publication, you may indicate that here to bypass the “Comments to the Author” section, enter your conflict of interest statement in the “Confidential to Editor” section, and submit your "Accept" recommendation.

Reviewer #1: All comments have been addressed

Reviewer #3: All comments have been addressed

2. Does this manuscript meet PLOS Global Public Health’s [publication criteria](https://journals.plos.org/globalpublichealth/s/criteria-for-publication)? Is the manuscript technically sound, and do the data support the conclusions? The manuscript must describe methodologically and ethically rigorous research with conclusions that are appropriately drawn based on the data presented.

Reviewer #1: Yes

Reviewer #3: Yes

3. Has the statistical analysis been performed appropriately and rigorously?

Reviewer #1: N/A

Reviewer #3: Yes

4. Have the authors made all data underlying the findings in their manuscript fully available (please refer to the Data Availability Statement at the start of the manuscript PDF file)?

The [PLOS Data policy](https://journals.plos.org/globalpublichealth/s/data-availability) requires authors to make all data underlying the findings described in their manuscript fully available without restriction, with rare exception. The data should be provided as part of the manuscript or its supporting information, or deposited to a public repository. For example, in addition to summary statistics, the data points behind means, medians and variance measures should be available. If there are restrictions on publicly sharing data—e.g. participant privacy or use of data from a third party—those must be specified.

Reviewer #1: Yes

Reviewer #3: Yes

5. Is the manuscript presented in an intelligible fashion and written in standard English?

PLOS Global Public Health does not copyedit accepted manuscripts, so the language in submitted articles must be clear, correct, and unambiguous. Any typographical or grammatical errors should be corrected at revision, so please note any specific errors here.

Reviewer #1: Yes

Reviewer #3: Yes

6. Review Comments to the Author

Please use the space provided to explain your answers to the questions above. You may also include additional comments for the author, including concerns about dual publication, research ethics, or publication ethics. (Please upload your review as an attachment if it exceeds 20,000 characters)

Reviewer #1:

I would consider reducing the images presented to make them more graphically readable. Others could be in the supplement.

Response:

We believe removing some images to supplementary files will make referencing them rather a little difficult. We will appropriate if they remain.

I would consider more specifically speak to how climate change will affect malaria predictions, as such predictions will look very different in the course of a decade. I would mention the phrase climate change in the paper.
<https://link.springer.com/article/10.1007/s00285-018-1229-7>

From before:
Climate Change. Although the model includes El Niño and weather information, I would still make note of how climate change which may disrupt current patterns and cause greater drought, seasonal changes, even population movement away from say dry areas to wetter areas, could affect predictions in the longerterm rather than just a year or so in advance. The modification of seasonal forcing functions could behave differently if climate change acerbated.

Response:

Changes have been on the statement above as in lines 559-566.

“Having modified the seasonal function of the application to reflect zonal malaria morbidity dynamics allows the application to be used to predict more accurately, the patterns and impact of various malaria intervention packages across different transmission settings in Ghana. However, assuming a constant rainfall and temperature is a limitation that could render the model to produce inaccurate results in the prediction window should the assumption fail. For instance if any of the zones experiences a severe draught or unusually high volumes of rainfall in the future as a result of climate change effects, this could impact on the predicted number of cases. Nevertheless, the forcing functions could be updated using locally reported weather data.”

I might make mention of varying socioeconomic levels across regions, as lower socioeconomic status and household wealth, which has been correlated with higher risk of malaria infection, in discussion.

Response:

The following statement has been added in lines 567-571 to address comment on the association of varying scoio-economic status on risk of infection

“Another limitation that arises is in not adjusting for the household socio-economic levels across that have not been accounted for these predicts. It has been shown that increase risk of malaria infection is highly associated with the wealth of the household [17]. The effect of the association on the impact of these interventions at the population level may however be averaged out”.

Would explain more why the intervention scenarios of health systems strengthening and strengthening the CHW/CHPS have significant overlaps. Would be more specific about what health systems strengthening means from a practical standpoint and why it would be a separate intervention scenario from CHW/CHPS strengthening

Response:

Health systems improvement statement edited to read….

“……………..It does seem, an increase in coverage with an accompanying health systems improvement by way of increased probability of seeking health and getting tested and treated once malaria is confirmed for all suspected cases leads to a more appreciable proportion of cases averted”

<https://www.ncbi.nlm.nih.gov/pmc/articles/PMC3120731/>
<https://www.ncbi.nlm.nih.gov/pmc/articles/PMC3483782/>

From before:
Check use of it's vs its
-> Response: edited appropriately

Reviewer #3: The manuscript elucidates the importance of bed nets, indoor insecticide spraying, and diagnosis and treatment of malaria as efficient intervention tools for modeling and forecasting the pre-elimination of malaria in Ghana. Although there are other significant intervention tools for modeling and forecasting. The writer believed that those mentioned are more appropriate and applicable to Ghana. It is a novel study in Ghana, but the writer still needs more references from similar studies to prove the genuineness of his findings. I could not see clearly any limitation to this study and further opportunities to explore.

Response:

Two limitations have been included as suggested on pp21-22. These are in the statements:

“………………..For instance if any of the zones experiences a severe draught or unusually high volumes of rainfall in the future as a result of climate change effects, this could impact on the predicted number of cases. Nevertheless, the forcing functions could be updated using locally reported weather data. Another limitation that arises is in not adjusting for the household socio-economic levels across that have not been accounted for these predicts. It has been shown that increase risk of malaria infection is highly associated with the wealth of the household [17]. The effect of the association on the impact of these interventions at the population level may however be averaged out.”

7. PLOS authors have the option to publish the peer review history of their article ([what does this mean?](https://journals.plos.org/globalpublichealth/s/editorial-and-peer-review-process#loc-peer-review-history)). If published, this will include your full peer review and any attached files.


**Do you want your identity to be public for this peer review?** If you choose “no”, your identity will remain anonymous but your review may still be made public.


For information about this choice, including consent withdrawal, please see our [Privacy Policy](https://www.plos.org/privacy-policy).

Reviewer #1: No

Reviewer #3: No
